# Green-Synthesized Silver Nanoparticles from *Filipendula ulmaria* and *Salvia verticillata* Extracts Exert Antimetastatic and Anti-Inflammatory Effects Through Redox-Mediated Nrf-2/NF-κB/MMP-2/9 Signaling in Human Colon Cancer Cells

**DOI:** 10.3390/antiox15081035

**Published:** 2026-08-19

**Authors:** Miloš Matić, Milica Paunović, Branka Ognjanović, Nikola Srećković, Nevena Mihailović, Vladimir Mihailović, Ana Obradović

**Affiliations:** 1 Department of Biology and Ecology, Faculty of Science, University of Kragujevac, 34000 Kragujevac, Serbia; milos.matic@pmf.kg.ac.rs (M.M.); milica.paunovic@pmf.kg.ac.rs (M.P.); branka.ognjanovic@pmf.kg.ac.rs (B.O.); 2 Department of Chemistry, Faculty of Science, University of Kragujevac, 34000 Kragujevac, Serbia; nikola.sreckovic@pmf.kg.ac.rs (N.S.); nevena.mihailovic@pmf.kg.ac.rs (N.M.)

**Keywords:** migrastatics, green-synthesized silver nanoparticles, colon cancer, oxidative/nitrosative stress, cell migration and invasiveness inhibition, Nrf-2/NF-κB/COX-2/MMP-2/9

## Abstract

Cancer metastasis, characterized by the dissemination of malignant cells from the primary tumor to distant organs, remains the leading cause of cancer-related mortality in solid tumors. In colorectal cancer (CRC), increasing attention has been directed toward therapeutic strategies aimed at suppressing cancer cell migration and invasion rather than solely reducing tumor mass, giving rise to the concept of migrastatic therapies. In the present study, green-synthesized silver nanoparticles (AgNPs), previously obtained using aqueous extracts of *Filipendula ulmaria* (L.) Maxim. and *Salvia verticillata* L., were evaluated for their antimigratory and anti-inflammatory potential in human colorectal carcinoma HCT-116 cells. Treatment with AgNPs induced considerable perturbations in cellular redox homeostasis, as evidenced by increased intracellular reactive oxygen species (ROS), lipid peroxidation (LPO), glutathione (GSH), and nitric oxide (NO) levels. These redox alterations were accompanied by a significant inhibition of cancer cell migration, together with reduced expression of matrix metalloproteinases MMP-2 and MMP-9, key mediators of extracellular matrix remodeling associated with tumor progression. AgNP exposure was associated with activation of the cytoprotective transcription factor Nrf-2 and suppression of the pro-inflammatory NF-κB/COX-2 signaling axis, indicating coordinated modulation of redox-sensitive pathways linked to tumor cell motility and inflammatory responses. Collectively, these findings demonstrate that green-synthesized AgNPs derived from *F. ulmaria* and *S. verticillata* exert multi-level regulatory effects on redox balance, inflammatory signaling, and migration-associated molecular markers in colorectal cancer cells. This study supports their potential as promising migrastatic nanocarriers for further investigation in colorectal cancer research.

## 1. Introduction

Colorectal cancer (CRC) is one of the most frequently diagnosed malignancies worldwide and represents a major health burden among gastrointestinal cancers. Despite significant advances in surgery, chemotherapy, and targeted therapies, therapy resistance and metastasis continue to represent major contributors to poor clinical outcomes, highlighting the urgent need for novel strategies targeting cancer progression and dissemination [1,2]. Although substantial progress has been achieved in anticancer therapy, no agents are currently approved specifically for the inhibition of metastatic disease progression [3,4].

Oxidative stress is a key contributor to tumor initiation and progression and occurs when the production of reactive species exceeds the capacity of antioxidant defense systems to maintain redox balance. Reactive oxygen and nitrogen species (ROS and RNS) act as key regulators of cell proliferation, migration, and immune responses. However, excessive accumulation of these species leads to oxidative and nitrosative stress, promoting genomic instability and tumor progression [5,6]. Redox-sensitive factors, including nuclear factor erythroid 2-related factor 2 (Nrf-2) and matrix metalloproteinases (MMP-2 and MMP-9), play critical roles in the regulation of antioxidant defense, extracellular matrix remodeling, and cancer cell migration [7].

Among the redox-regulated transcription factors, Nrf-2 and nuclear factor kappa B (NF-κB) play central and often opposing roles in cellular signaling. Nrf-2 primarily regulates antioxidant and cytoprotective responses, although its persistent activation in cancer cells may also contribute to tumor survival and therapy resistance [8,9]. In contrast, NF-κB is a central regulator of inflammation, proliferation, and metastasis, and its constitutive activation is strongly associated with colorectal cancer progression [10,11,12]. Dysregulation of the redox–inflammatory axis promotes oxidative stress adaptation, chronic inflammation, and enhanced cancer cell motility, making it an important therapeutic target [13,14].

Natural products, particularly bioactive constituents of medicinal plants, have gained increasing attention as promising anticancer agents because of their ability to modulate multiple signaling pathways involved in tumor growth, inflammation, and metastasis, often with lower systemic toxicity compared to conventional chemotherapeutics [15]. In parallel, the integration of nanotechnology with plant-derived compounds has enabled the development of green-synthesized nanomaterials with improved biological activity and eco-friendly production methods [16]. Nanomaterials used in cancer therapy are often referred to as targeted therapeutic systems, as they can enhance drug delivery, improve drug accumulation in tumor tissue, and reduce systemic toxicity [17].

Silver nanoparticles (AgNPs) have attracted considerable attention in biomedical research due to their broad biological activities, including anticancer effects. Green synthesis is one of the most widely investigated approaches to produce AgNPs because it avoids the use of hazardous reducing and stabilizing agents while enabling the fabrication of biocompatible nanomaterials. Plant extracts are particularly attractive for nanoparticle synthesis due to their rich phytochemical composition, including flavonoids, phenolic acids, polyphenols, terpenoids, alkaloids, and other secondary metabolites, which simultaneously reduce Ag^+^ ions and stabilize the resulting nanoparticles. Consequently, the physicochemical characteristics and biological activity of green-synthesized AgNPs strongly depend on the plant species and its phytochemical profile, as well as on nanoparticle size, morphology, and surface chemistry [18,19].

Numerous studies have demonstrated that plant-mediated AgNPs exhibit pronounced antimicrobial, antioxidant, anti-inflammatory, and anticancer activities, often exceeding those of conventionally synthesized nanoparticles due to the bioactive phytochemical corona surrounding the silver core [20,21]. Current evidence suggests that the biological activity of green-synthesized AgNPs results from the combined contribution of the silver core and the plant-derived capping molecules, whose synergistic interactions influence nanoparticle stability, cellular uptake, and biological responses [22]. Therefore, selecting appropriate plant species for nanoparticle synthesis represents an important strategy for tailoring their biological properties and expanding their potential biomedical applications. Green-synthesized AgNPs have been reported to exert anticancer effects through multiple mechanisms, including ROS generation, apoptosis induction, DNA damage, cell cycle disruption, and the modulation of inflammatory and migratory signaling pathways [23,24]. Consequently, an increasing number of studies are focusing on understanding how different medicinal plants influence the therapeutic potential of green-synthesized AgNPs, particularly in cancer-related applications [25,26]. However, the molecular basis of these plant-dependent biological effects remains largely unexplored.

*Filipendula ulmaria* (L.) Maxim. is traditionally used for the treatment of inflammatory disorders and is rich in phenolic compounds such as gallic, ellagic, and salicylic acids, as well as flavonoids and tannins [27,28]. *Salvia verticillata* L. contains rosmarinic and caffeic acid derivatives and exhibits strong antioxidant and anti-inflammatory properties [29,30,31]. In addition, both plant extracts have been reported to possess strong antioxidant activity, reflecting their high content of redox-active phenolic compounds capable of reducing metal ions and stabilizing the resulting nanoparticles [28,30]. These characteristics make them promising candidates for the green synthesis of silver nanoparticles with potential biological activity. Furthermore, differences in their phytochemical composition may influence the physicochemical characteristics and biological properties of the synthesized AgNPs. In our previous study, silver nanoparticles synthesized using aqueous extracts of *F. ulmaria* (FUAgNPs) and *S. verticillata* (SVAgNPs) demonstrated significant antiproliferative effects against colon cancer cells [32]. Green synthesis yielded silver nanoparticles with nanoscale dimensions and a well-defined crystalline silver structure, while phytochemicals originating from the plant extracts were retained on the nanoparticle surface [32].

Building on these findings, the present study aims to elucidate the molecular mechanisms underlying the anticancer activity of these green-synthesized AgNPs in HCT-116 colorectal cancer cells, with a particular focus on redox homeostasis, oxidative and nitrosative stress, cell migration, MMP-2/9 activity, and the modulation of inflammation via the Nrf-2/NF-κB/COX-2 signaling axis [33].

## 2. Materials and Methods

### 2.1. Cell Culture and Treatment

The human colorectal carcinoma cell line HCT-116 (ATCC, Manassas, VA, USA) was cultured in Dulbecco’s modified Eagle medium (DMEM) supplemented with 10% fetal bovine serum (FBS), 100 IU/mL penicillin, and 100 µg/mL streptomycin at 37 °C in a humidified atmosphere containing 5% CO_2_.

Cells were seeded into appropriate culture plates at approximately 80% confluence and allowed to adhere for 24 h before treatment. Silver nanoparticles (AgNPs) used in this study were synthesized by a green approach using aqueous extracts of aerial parts of *S. verticillata* (SVAgNPs) and *F. ulmaria* (FUAgNPs), as previously described [32]. Briefly, the nanoparticles were obtained under optimized synthesis conditions and characterized by UV–Vis spectroscopy, Fourier transform infrared spectroscopy (FTIR), X-ray diffraction (XRD), dynamic light scattering (DLS), and scanning electron microscopy coupled with energy-dispersive spectroscopy (SEM/EDS). Both types of AgNPs exhibited a crystalline structure, with silver as the dominant constituent in their structure. According to the FTIR analysis, absorption bands corresponding to functional groups of phytochemicals were detected in both the plant extracts and AgNP spectra, indicating that plant-derived compounds remained adsorbed on the surface of the AgNPs. DLS analysis showed that the majority of nanoparticles had diameters ranging from 40 to 70 nm. The previously characterized nanoparticles were directly used for biological assays without additional modification. Silver nanoparticles (FUAgNPs and SVAgNPs) were applied at concentrations of 5–100 µg/mL depending on the assay. The concentrations used for migration, MMP-related, and inflammatory signaling assays were selected based on our previously published cytotoxicity (IC_50_) data and preliminary redox analyses, aiming to evaluate mechanistic effects at biologically relevant concentrations while avoiding excessive non-specific cellular damage. Untreated cells served as the control group. All experiments were performed in triplicate.

### 2.2. Preparation of Cell Samples for Oxidative Stress Assays

After 24 or 72 h of treatment, HCT-116 cells were harvested by trypsinization, collected by centrifugation (1000× *g*, 10 min, 4 °C), and the culture medium was discarded. The cell pellet was washed once with ice-cold phosphate-buffered saline (PBS) and centrifuged again under the same conditions. Subsequently, the cell suspension was adjusted to a final concentration of 1 × 10^5^ cells/mL, and the standardized samples were used for the determination of superoxide anion radical (O_2_^•−^), nitric oxide (NO), and reduced and total glutathione (GSH and tGSH) levels.

### 2.3. Determination of Superoxide Anion Radical (NBT Assay)

Superoxide anion radical (O_2_^•−^) production was assessed using the nitro blue tetrazolium (NBT) reduction assay [34]. Following treatment, NBT solution (5 mg/mL, 20 µL) was added to samples and incubated for 1 h at 37 °C. The formed formazan crystals were dissolved in DMSO, and absorbance was measured at 550 nm using a microplate reader. Superoxide anion radical levels were calculated based on a standard curve and normalized to the number of viable cells. Results were expressed as nmol per 10^5^ cells.

### 2.4. Determination of Nitric Oxide Levels (Griess Assay)

Nitric oxide production was evaluated indirectly by measuring nitrite accumulation using the Griess reaction according to the previously described method [35]. Equal volumes of sulfanilamide (1%) and N-(1-naphthyl) ethylenediamine dihydrochloride (0.1%) were added, followed by incubation for 10 min at room temperature in the dark. Absorbance was measured at 550 nm using a microplate reader. Nitrite concentrations were calculated based on a standard curve, normalized to the number of viable cells, and results were expressed as µmol per 10^5^ cells.

### 2.5. Determination of Reduced and Total Glutathione Levels

Reduced glutathione (GSH) and total glutathione levels were determined based on its reaction with 5,5′-dithiobis-(2-nitrobenzoic acid) (DTNB), resulting in the formation of the yellow-colored product 5′-thio-2-nitrobenzoic acid (TNB) [36,37].

Following treatment, cells were precipitated with 2.5% sulfosalicylic acid and lysed by sonication. The obtained samples were incubated with a reaction mixture containing DTNB in 100 mM phosphate buffer for the determination of reduced glutathione (GSH). For total glutathione determination, the same reaction mixture was supplemented with 1 mM NADPH and 0.7 U glutathione reductase. Absorbance was measured at 405 nm using a microplate reader. Glutathione concentrations were calculated based on a standard curve, normalized to the number of viable cells, and results were expressed as µmol per 10^5^ cells.

### 2.6. Determination of Lipid Peroxidation (TBARS Assay)

Lipid peroxidation was determined by measuring thiobarbituric acid-reactive substances (TBARS) [38]. HCT-116 cells were treated with FUAgNP and SVAgNP at concentrations of 5 µg/mL and 50 µg/mL. Malondialdehyde (MDA), a marker of lipid peroxidation, was quantified by reaction with thiobarbituric acid at 95 °C. Absorbance was measured at 535 nm using a microplate reader. MDA concentrations were calculated based on a standard curve, normalized to total protein content, and results were expressed as nmol MDA/mg protein.

### 2.7. Cell Migration Assay (Transwell Method)

Cell migration was evaluated using Transwell chambers (8 µm pore size; Greiner Bio-One, St. Gallen, Switzerland) as previously described [39]. HCT-116 cells were treated with AgNPs at concentrations of 5 µg/mL and 50 µg/mL.

After treatment, 1 × 10^5^ cells in 500 µL of serum-free medium were seeded into the upper chamber, while 750 µL of DMEM supplemented with 10% FBS was added to the lower chamber. Following incubation, non-migrated cells were removed from the upper surface of the membrane.

Migrated cells were fixed with 4% paraformaldehyde and stained with 0.1% crystal violet. The membranes were transferred to 96-well plates, and the dye was solubilized in 10% acetic acid. Absorbance was measured at 595 nm using a microplate reader. Migration was expressed as a percentage relative to control cells.

### 2.8. ELISA-Based Protein Determination (Nrf-2, MMP-2, MMP-9, NF-κB, COX-2)

Protein levels of Nrf-2, NF-κB, COX-2, MMP-2, and MMP-9 were determined using commercial ELISA kits (Elabscience, Houston, TX, USA) according to the manufacturer’s instructions and previously described methods [23]. HCT-116 cells were treated with FUAgNP and SVAgNP (5 and 50 µg/mL) for 72 h. After treatment, cells were collected, washed with phosphate-buffered saline (PBS), and lysed by sonication. Cell lysates were centrifuged, and the supernatants were used for analysis. Absorbance was measured at 450 nm using a microplate reader. Concentrations were calculated from a standard calibration curve. The obtained values were normalized to the total protein content determined by the Bradford assay and expressed as ng/mg protein.

### 2.9. Determination of Total Protein Content

Total protein concentration was determined using the Bradford assay according to the method described by Bradford [40]. Briefly, cell lysates were prepared in an appropriate lysis buffer, and aliquots of each sample were mixed with Bradford reagent. After incubation at room temperature for 5–10 min, absorbance was measured at 595 nm using a microplate reader. Protein concentrations were calculated based on a standard curve generated using bovine serum albumin (BSA) and were used for normalization of the ELISA-based measurements.

### 2.10. Quantitative Real-Time PCR (qRT-PCR)

Total RNA was isolated from HCT-116 cells using TRIzol reagent according to the method described by Chomczynski and Sacchi [41]. RNA purity and concentration were assessed spectrophotometrically (A260/A280 ≥ 1.8).

cDNA was synthesized from 2 µg of total RNA using Superscript II reverse transcriptase (Invitrogen, Carlsbad, CA, USA) in a total volume of 20 µL. Quantitative PCR was performed using SYBR Green PCR Master Mix (Applied Biosystems, Foster City, CA, USA) in a final reaction volume of 20 µL containing 0.5 µM primers and 2 µL cDNA.

β-Actin forward primer 5’-GTTCTCAAGGCACAGGTCTC-3’,

reverse primer 5’-GGGAGACCAAAGCCTTCAT-3’,

MMP-9 forward primer 5’-ACCTCGAACTTTGACAGCGACA-3’,

reverse primer 5’-GATGCCATTCACGTCGTCCTTA-3’ [42].

Amplification was performed on a 7500 Real-Time PCR System (Thermo Fisher Scientific, Waltham, MA, USA) under the following conditions: 95 °C for 3 min, followed by 40 cycles of 95 °C for 15 s and 55 °C for 60 s.

The obtained results were analyzed by the 2^−ΔΔCt^ method [43]. The negative control samples (without cDNA) were also amplified in qPCR reaction to confirm that the contamination with genomic DNA was in the acceptable range [44]. The results were expressed as the percentage of the change compared to control values.

### 2.11. Statistical Analysis

Data are presented as the mean ± standard error of the mean (SEM) from at least three independent biological experiments. Statistical analysis was performed using one-way ANOVA followed by Tukey’s post hoc test (IBM SPSS Statistics 23). Differences were considered statistically significant at *p* < 0.05.

## 3. Results

### 3.1. Redox Status in HCT-116 Cells

The effects of FUAgNP and SVAgNP were evaluated in HCT-116 human colorectal cancer cells at concentrations ranging from 5 to 100 µg/mL. To assess changes in cellular redox homeostasis, levels of superoxide anion (O_2_^•−^), nitric oxide (NO_2_^−^), glutathione (GSH), lipid peroxidation, and Nrf-2 expression were measured.

#### 3.1.1. Superoxide Anion Radical (NBT Assay)

**Figure 1:**
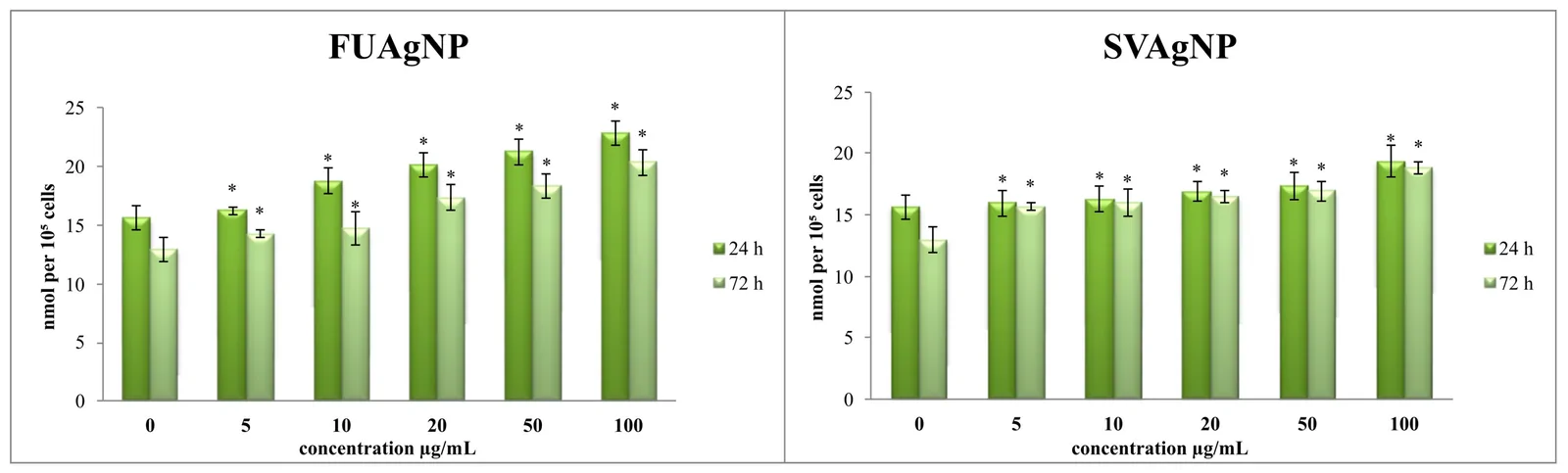
The level of O_2_^•–^ in HCT-116 cells after 24 and 72 h of treatment with FUAgNP and SVAgNP. Data are presented as the mean ± standard error of the mean (SEM). * *p* < 0.05 vs. the control group.

Treatment with FUAgNP and SVAgNP significantly increased the O_2_^•−^levels in HCT-116 cells compared to the control group at both 24 h and 72 h exposure periods (Figure 1). These findings demonstrate that both AgNP formulations induced a pro-oxidative shift in intracellular redox status through increased superoxide anion radical production, which may contribute to the biological effects observed after nanoparticle treatment.

#### 3.1.2. Nitrite Levels (Griess Assay)

**Figure 2:**
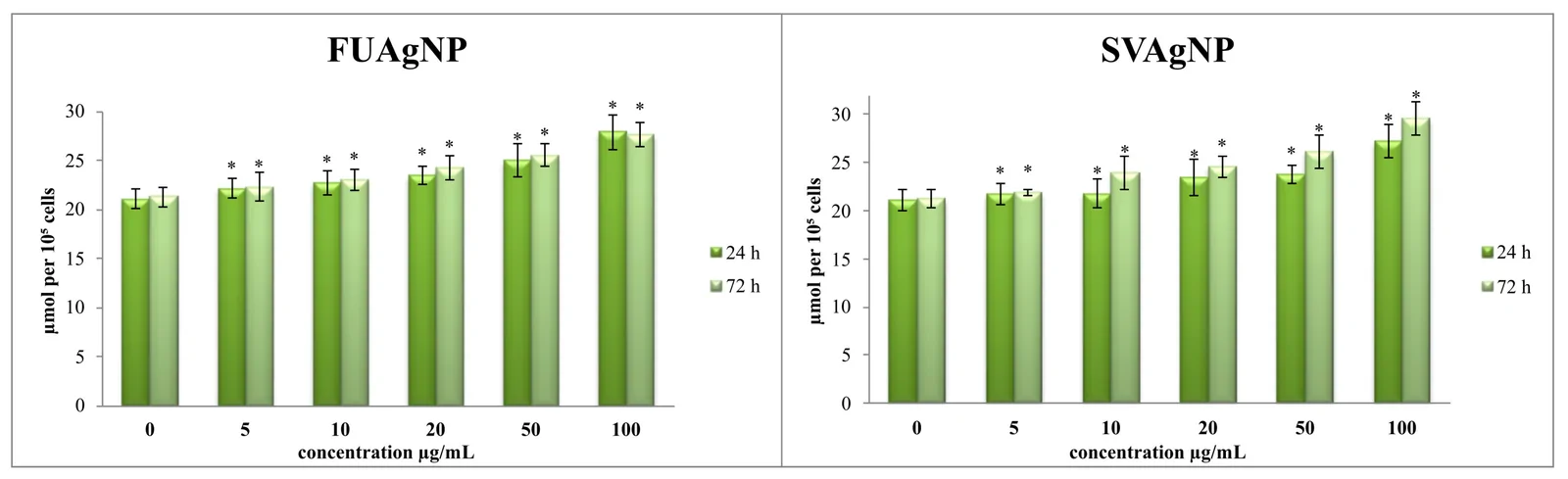
The level of NO_2_^−^ in HCT-116 cells after 24 and 72 h of treatment with FUAgNP and SVAgNP. Data are presented as the mean ± standard error of the mean (SEM). * *p* < 0.05 vs. the control group.

Treatment with FUAgNP and SVAgNP significantly increased the nitrite (NO_2_^−^) levels in HCT-116 cells compared with the control group at both 24 h and 72 h exposure periods (Figure 2). These findings indicate that both AgNP formulations altered the intracellular nitrosative status following nanoparticle treatment. Increased nitrite accumulation was observed after exposure to the tested nanoparticle concentrations, indicating enhanced NO-related responses in the treated HCT-116 cells.

#### 3.1.3. Reduced and Total Glutathione

**Figure 3:**
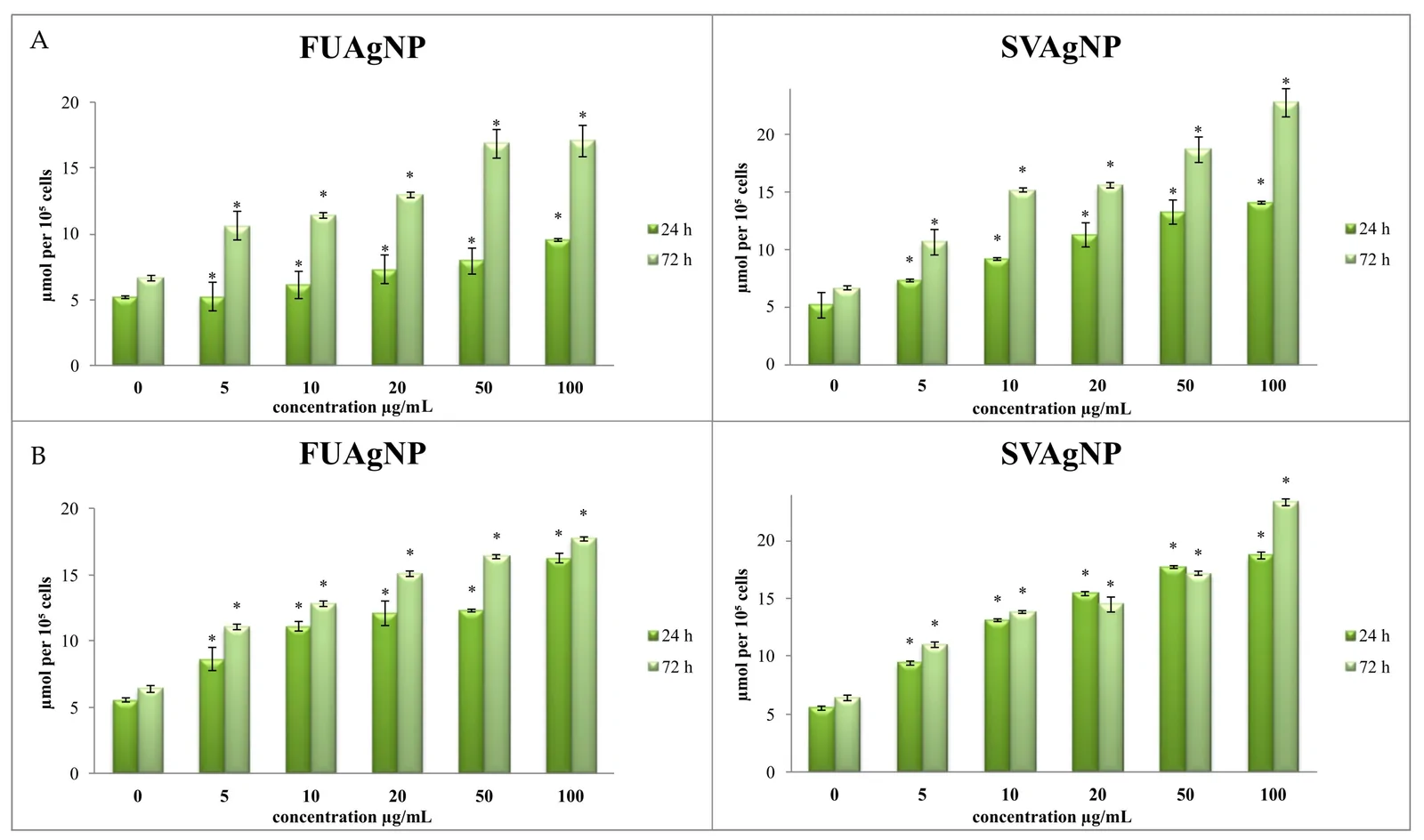
The level of (**A**) reduced glutathione and (**B**) total glutathione in HCT-116 cells after 24 and 72 h of treatment with FUAgNP and SVAgNP. Data are presented as the mean ± standard error of the mean (SEM). * *p* < 0.05 vs. the control group.

The effects of FUAgNP and SVAgNP treatment on the reduced and total glutathione (GSH and tGSH) levels in HCT-116 cells are presented in Figure 3. Both AgNP formulations significantly increased the GSH and tGSH levels compared with the untreated control group. The observed elevation in glutathione levels was detected after both short-term (24 h) and long-term (72 h) exposure, indicating that FUAgNP and SVAgNP induced changes in cellular antioxidant defense mechanisms at both analyzed time points. Differences in the magnitude of the response between exposure periods were observed, reflecting the dynamic regulation of intracellular redox balance following nanoparticle treatment.

#### 3.1.4. Lipid Peroxidation

**Figure 4:**
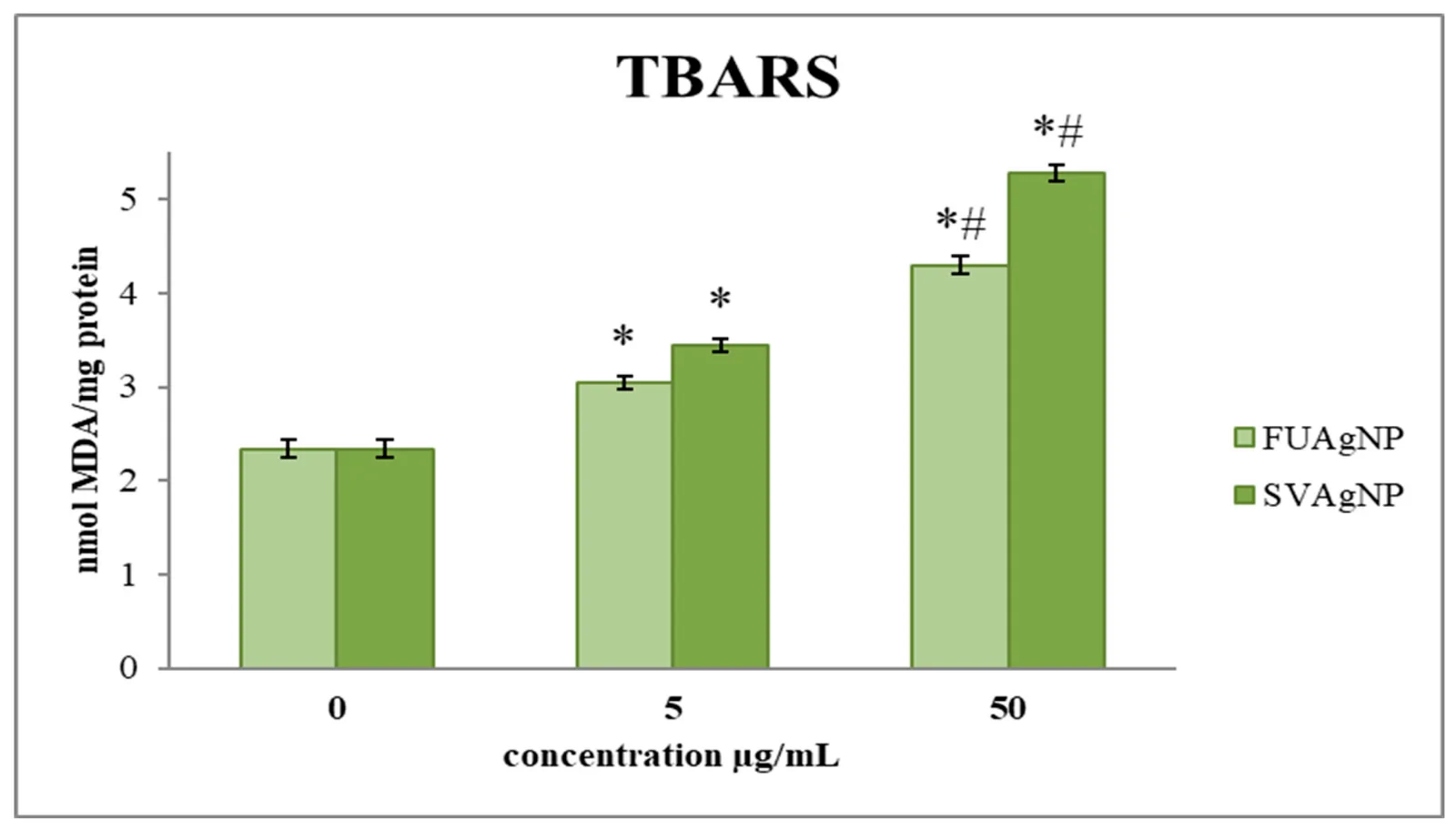
The level of MDA in HCT-116 cells after 72 h of treatment with FUAgNP and SVAgNP. Data are presented as the mean ± standard error of the mean (SEM). * *p* < 0.05 compared with the control group; # *p* < 0.05 between different concentrations of the same AgNP treatment.

The effects of FUAgNP and SVAgNP on lipid peroxidation in HCT-116 cells were assessed by measuring the malondialdehyde (MDA) levels (Figure 4). After 72 h of treatment, MDA concentrations were significantly increased in the AgNP-treated cells compared with the untreated control cells. In both the FUAgNP- and SVAgNP-treated cells, the 50 µg/mL concentration resulted in significantly higher MDA levels compared with the 5 µg/mL concentration, indicating a stronger induction of lipid peroxidation at the higher tested concentration. Lipid peroxidation generates reactive aldehydes, such as MDA, which can modify proteins, DNA, and other biomolecules, altering their structure and function. This process has been implicated in tumor promotion, as reactive lipid-derived metabolites can contribute to oxidative stress and cellular damage. Measurement of MDA levels has been widely employed in cancer research, including studies on colorectal cancer, to evaluate oxidative damage and its potential role in tumor development [45].

#### 3.1.5. Nrf-2 Protein Levels

**Figure 5:**
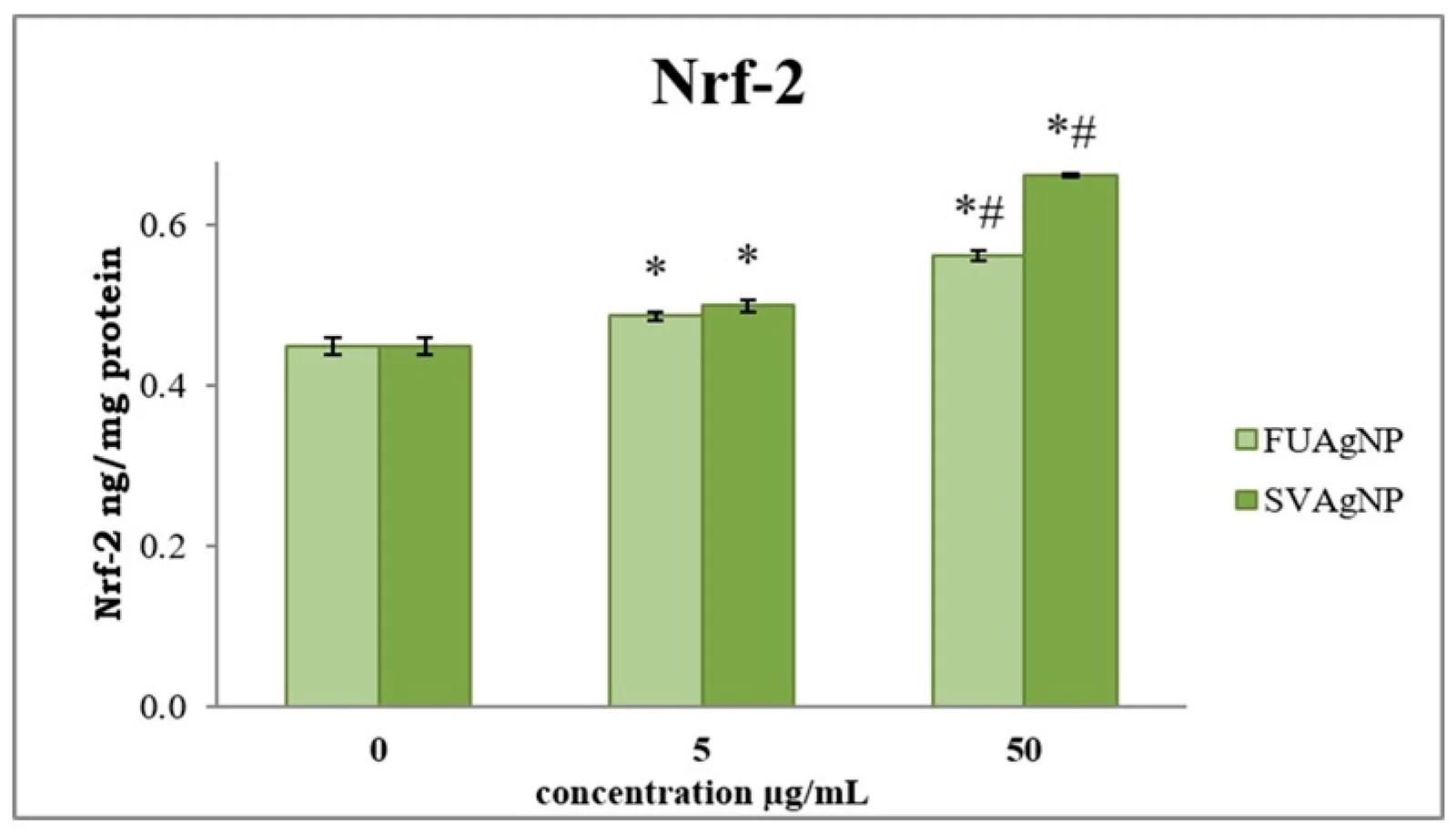
The level of Nrf-2 in HCT-116 cells after 72 h of treatment with FUAgNP and SVAgNP. Data are presented as the mean ± standard error of the mean (SEM). * *p* < 0.05 compared with the control group; # *p* < 0.05 between different concentrations of the same AgNP treatment.

Treatment of HCT-116 cells with FUAgNP and SVAgNP at both tested concentrations significantly increased the Nrf-2 levels compared with the untreated control group (Figure 5). In both the FUAgNP- and SVAgNP-treated cells, the increase in Nrf-2 levels was more pronounced at 50 µg/mL compared with 5 µg/mL, and the difference between concentrations reached statistical significance.

These findings indicate that the higher tested concentration induced a stronger modulation of Nrf-2 expression under the applied experimental conditions. The observed increase in Nrf-2 levels was accompanied by alterations in intracellular redox parameters, including increased ROS and nitrite levels, suggesting activation of cellular redox-regulatory responses following AgNP treatment.

### 3.2. Effects of FUAgNP and SVAgNP on Cell Migration and MMP Expression

To evaluate the effects of FUAgNP and SVAgNP on the invasive potential of HCT-116 cells, cell migration, MMP-2 and MMP-9 protein levels, and MMP-9 gene expression were analyzed following nanoparticle treatment.

#### 3.2.1. Cell Migration (Transwell Assay)

**Figure 6:**
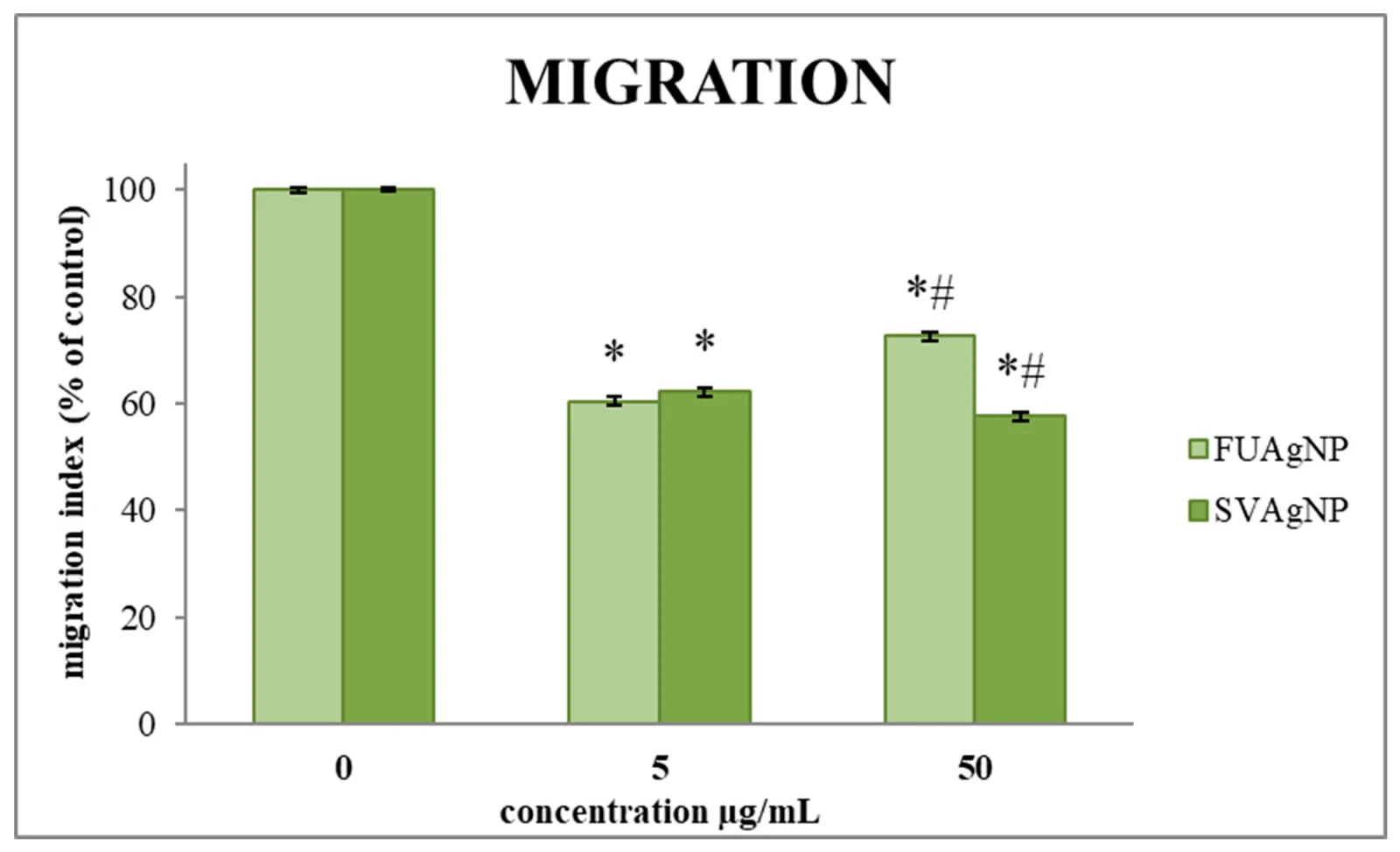
Cell migration of HCT-116 cells after 72 h of treatment with FUAgNP and SVAgNP. Data are presented as the mean ± standard error of the mean (SEM). * *p* < 0.05 compared with the control group; # *p* < 0.05 between different concentrations of the same AgNP treatment.

The effects of FUAgNP and SVAgNP on the migratory capacity of HCT-116 cells were evaluated using a 2D Transwell migration assay. Both nanoparticle formulations significantly reduced the migratory capacity of HCT-116 cells compared with the untreated control group (Figure 6). Although differences between the tested concentrations were observed, the magnitude of the inhibitory effect varied between the two nanoparticle formulations. SVAgNP treatment reduced cell migration to approximately 57% of the control value, corresponding to an inhibition of about 43%, with the most pronounced effect observed at 50 µg/mL.

#### 3.2.2. MMP-2 and MMP-9 Levels

**Figure 7:**
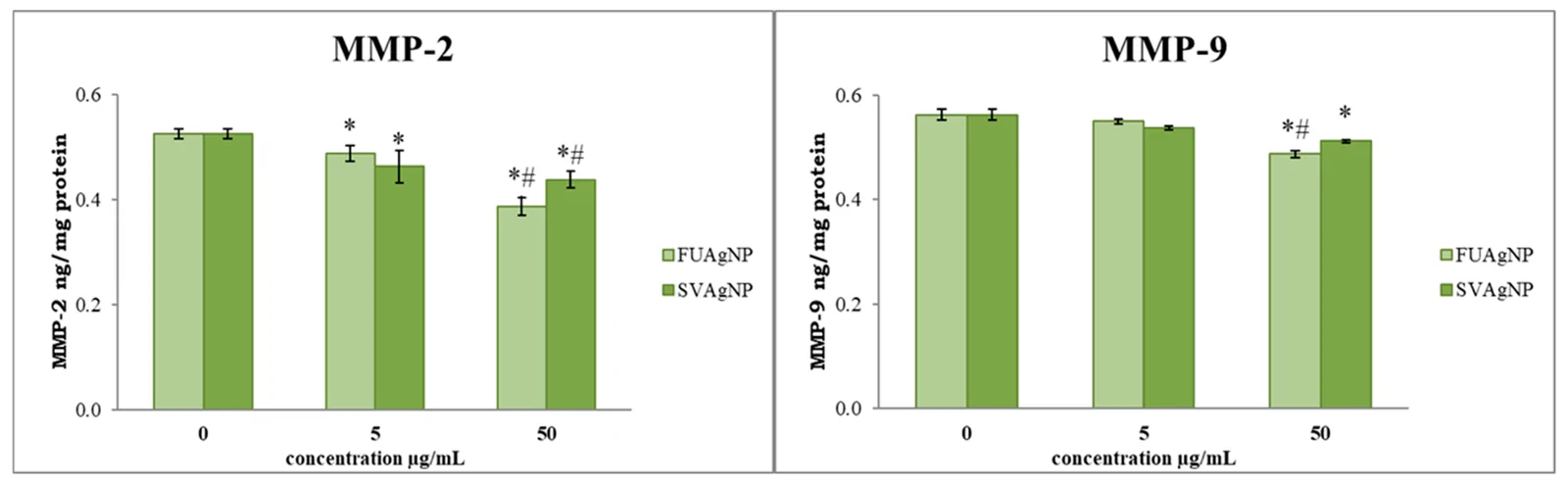
MMP-2 and MMP-9 expression levels in HCT-116 cells following 72 h of treatment with FUAgNP and SVAgNP. Data are presented as the mean ± standard error of the mean (SEM). * *p* < 0.05 compared with the control group; # *p* < 0.05 between different concentrations of the same AgNP treatment.

Treatment with both FUAgNP and SVAgNP significantly reduced the MMP-9 protein levels compared with the control group, with the most pronounced decrease observed after 72 h exposure to 50 µg/mL FUAgNP (Figure 7). The MMP-2 levels also decreased following treatment with both nanoparticle formulations, although the magnitude of the changes was less pronounced than that observed for MMP-9. Overall, both metalloproteinases exhibited lower protein levels in the treated cells compared with the control group. The observed reductions in MMP-2 and MMP-9 protein levels were accompanied by decreased cell migration and alterations in intracellular redox parameters, including increased ROS and nitrite levels.

#### 3.2.3. Relative Expression of MMP-9

**Figure 8:**
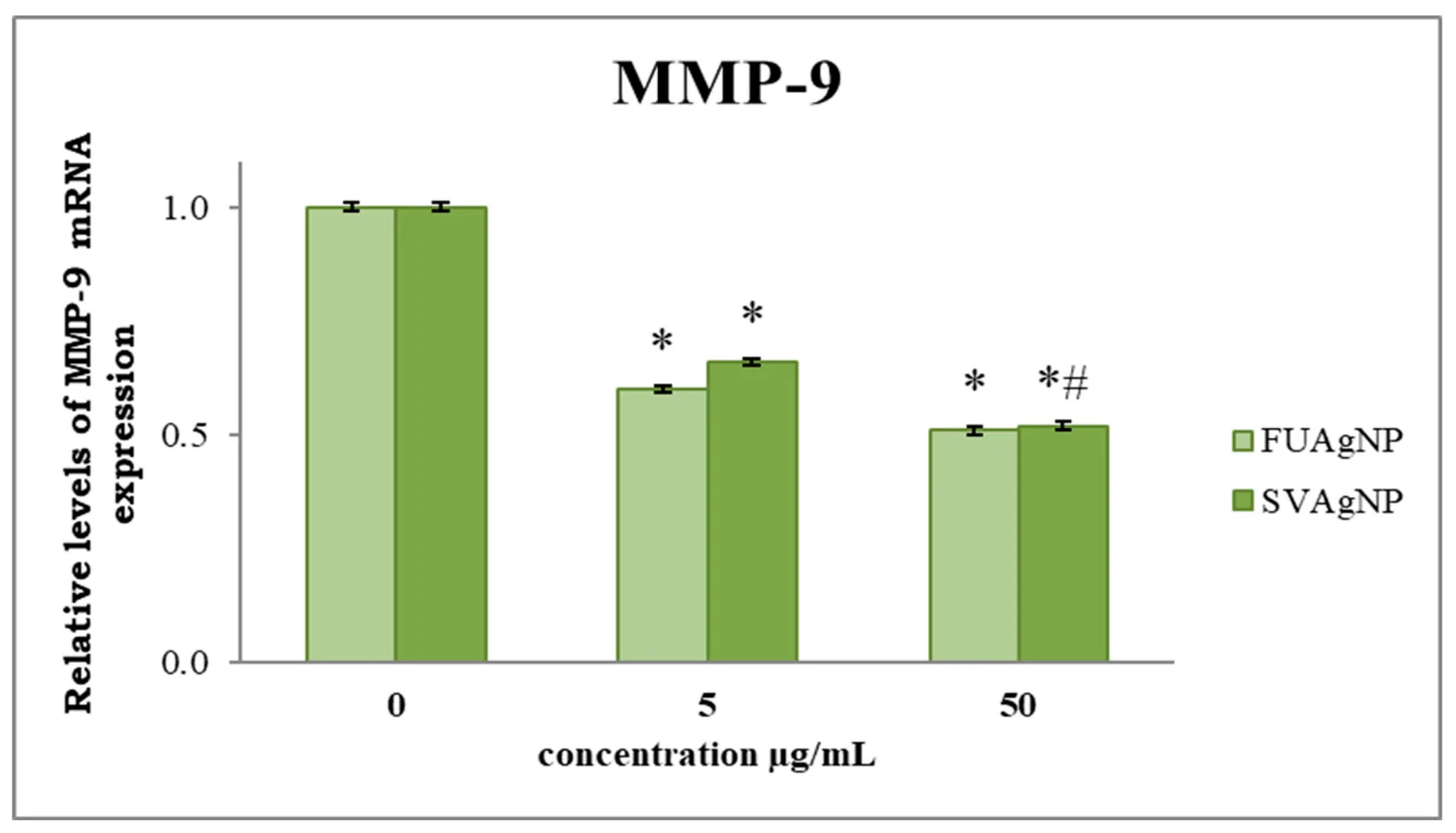
Relative mRNA expression levels of MMP-9 in HCT-116 cells following 72 h of treatment with FUAgNP and SVAgNP. Data are presented as the mean ± standard error of the mean (SEM). * *p* < 0.05 compared with the control group; # *p* < 0.05 between different concentrations of the same AgNP treatment.

To further evaluate the effects of FUAgNP and SVAgNP on the invasive potential of HCT-116 cells, the relative mRNA expression of MMP-9 was analyzed. Treatment with both AgNP types at the tested concentrations significantly reduced the MMP-9 mRNA levels compared with the control cells (Figure 8).

This transcriptional downregulation is consistent with the observed decreases in MMP-9 protein levels, reduced cell migration, and elevated ROS and nitrite production. These results suggest that FUAgNP and SVAgNP modulate cancer cell invasiveness at both the transcriptional and proteolytic levels, highlighting a coordinated effect on the metastatic phenotype.

### 3.3. Effects of FUAgNP and SVAgNP on Inflammatory Mediators

To investigate the effects of FUAgNP and SVAgNP on inflammatory signaling in HCT-116 cells, the levels of the pro-inflammatory mediators NF-κB and COX-2 were evaluated following nanoparticle treatment.

#### 3.3.1. NF-κB Protein Levels

**Figure 9:**
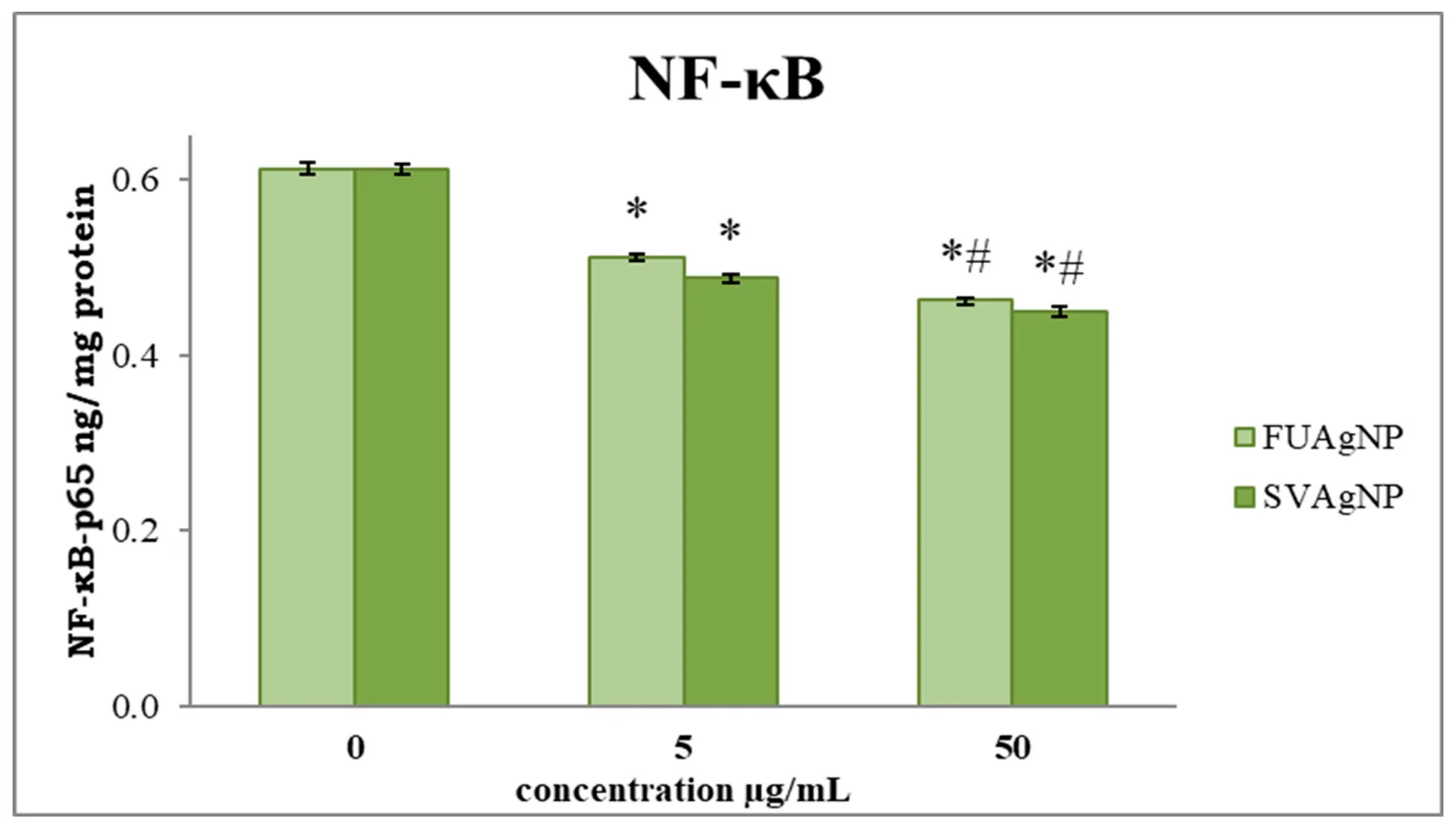
NF-κB protein levels in HCT-116 cells following 72 h of treatment with FUAgNP and SVAgNP. Data are presented as the mean ± standard error of the mean (SEM). * *p* < 0.05 compared with the control group; # *p* < 0.05 between different concentrations of the same AgNP treatment.

NF-κB is a key transcription factor involved in the regulation of inflammatory responses, cell survival, and tumor progression. Treatment with FUAgNP and SVAgNP significantly decreased the NF-κB protein levels in HCT-116 cells at both tested concentrations compared with the untreated control group (Figure 9). In both the FUAgNP- and SVAgNP-treated cells, NF-κB levels were significantly lower at 50 µg/mL than at 5 µg/mL, indicating a more pronounced effect at the higher concentration. Reduced NF-κB levels were observed together with decreased cell migration and lower MMP-2 and MMP-9 protein levels following nanoparticle treatment.

#### 3.3.2. COX-2 Levels

COX-2 is an inducible pro-inflammatory enzyme involved in tumor progression, angiogenesis, invasion, and metastasis. Increased COX-2 expression is frequently associated with colorectal cancer progression and poor prognosis.

**Figure 10:**
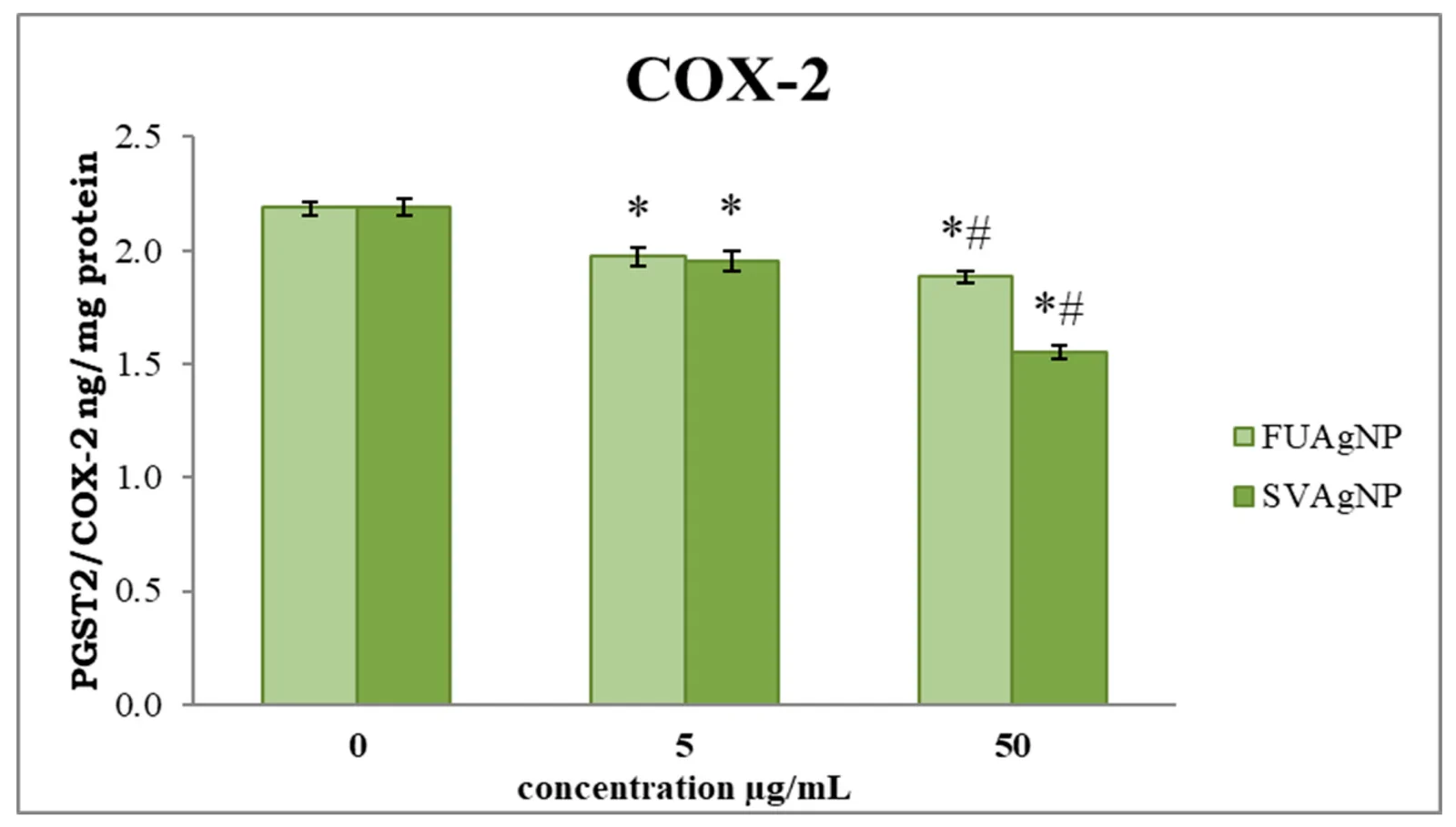
COX-2 protein levels in HCT-116 cells following 72 h of treatment with FUAgNP and SVAgNP. Data are presented as the mean ± standard error of the mean (SEM). * *p* < 0.05 compared with the control group; # *p* < 0.05 between different concentrations of the same AgNP treatment.

Treatment of HCT-116 cells with FUAgNP and SVAgNP for 72 h significantly decreased the COX-2 levels compared to the untreated control cells (Figure 10). Both types of AgNPs reduced COX-2 expression at the tested concentrations, indicating the suppression of inflammatory signaling pathways in colorectal cancer cells. The observed reduction in COX-2 levels was accompanied by decreased NF-κB activity, inhibition of cell migration and invasion, and downregulation of MMP-2 and MMP-9.

## 4. Discussion

Colorectal cancer progression is strongly associated with metastatic dissemination driven by the increased migration and invasion of malignant cells [46]. Green-synthesized silver nanoparticles (AgNPs) have attracted considerable attention as promising anticancer agents due to their biocompatibility and the presence of plant-derived phytochemicals on their surface, which may synergistically enhance their biological activity [47,48]. In our previous study, FUAgNPs and SVAgNPs exhibited significant antiproliferative activity against HCT-116 colorectal cancer cells [32]. Building on these findings, the present study investigated the molecular mechanisms underlying their antitumor and antimigratory effects, with particular emphasis on redox homeostasis, inflammatory signaling, and the regulation of migration-associated markers.

In agreement with the previous reports, our results showed that both FUAgNP and SVAgNP significantly increased the O_2_^•−^ levels in HCT-116 cells, indicating strong prooxidative activity, particularly after short-term exposure, possibly due to the required time delay for the activation of cell protection mechanisms. Excessive ROS production can overwhelm these adaptive responses, leading to oxidative damage and cytotoxicity [49]. Also, nitrite levels (NO_2_^−^) were significantly elevated, indicating the upregulation of NO-mediated cellular effects. The results show increased levels of LPO, suggesting strong free radical impact on cell membranes. These findings together confirm that the tested nanoparticles unequivocally generate a prooxidative state in cancer cells affecting ROS-dependent signaling pathways.

In the present study, the treatment with nanoparticles led to the significant increase in NO levels, accompanied by a reduction in tumor cell migratory capacity. The combined modulation of ROS and NO signaling likely contributes to the coordinated regulation of cytoskeletal organization, cell adhesion, and motility [50,51]. These observations suggest that enhanced NO bioavailability may contribute to the antimigratory effects of the tested nanoparticles through the regulation of signaling pathways affecting cell motility [52].

The recorded upregulation of Nrf-2 after treatment may represent a compensatory cellular response to nanoparticle-induced redox imbalance. The recruitment of antioxidative response in HCT-116 cells is further supported by the concomitant elevation of intracellular glutathione levels, the probable Nrf-2 downstream signaling target. Since glutathione serves as a key indicator of intracellular redox status [53], the elevated reduced glutathione levels indicate the possible activation of glutathione biosynthesis pathways induced by Nrf-2 detected in the study [54]. The increased levels of nitric oxide (NO) could activate the Nrf-2 activation pathway by inducing the S-nitrosation or oxidation of Keap1, an inhibitor of Nrf-2. This modification induces Keap1 detachment, nuclear translocation of Nrf-2 and transcription of various protective antioxidants and detoxifying genes [55,56]. Furthermore, nitric oxide exhibits significant anti-inflammatory potential in numerous physiological compartments [57].

Tumor cell migration is a key determinant of metastasis. Both FUAgNP and SVAgNP significantly suppressed HCT-116 migration. This effect correlated with reduced MMP-2 and MMP-9 protein levels, as well as decreased MMP-9 mRNA expression, highlighting that AgNPs can attenuate their transcription rate and protein content [58,59]. The 72 h treatment period reflects an integrated cellular response in which transcriptional regulation, protein synthesis, secretion, and protein degradation occur at different rates. Therefore, discrepancies between MMP-9 mRNA expression and protein abundance at the same time point are expected and reflect the complex, multilayered regulation of MMP-9 expression and activity.

Although MMP-9 mRNA expression and protein levels exhibited a similar inhibitory trend following FUAgNP and SVAgNP treatment, the magnitude of the changes was not identical. This difference likely reflects the distinct regulatory mechanisms governing gene and protein expression. While mRNA levels primarily indicate transcriptional regulation, protein abundance is additionally influenced by mRNA stability, translation efficiency, intracellular processing, secretion, extracellular activation, and protein turnover [60]. Furthermore, MMP-9 is subject to extensive post-transcriptional and post-translational regulation, which may further contribute to differences between transcript and protein levels measured at the same time point [61].

Therefore, the observed decrease in MMP-9 protein levels together with reduced MMP-9 mRNA expression after 72 h of treatment suggests that FUAgNP and SVAgNP affect MMP-9 regulation at both the transcriptional and protein levels, while the difference in the extent of changes reflects the complexity of MMP-9 regulation in cancer cells.

Similar antimetastatic effects of plant-derived AgNPs have been reported in HepG2 and breast cancer cell models, reinforcing the role of MMP modulation in nanoparticle-mediated inhibition of tumor invasion [23,62]. Tumor cells frequently activate Nrf-2-dependent antioxidant pathways as a protective mechanism in response to oxidative and/or nitrosative stress. Studies indicate that the activation of NF-κB signaling significantly contributes to cell proliferation, migration, and invasion capacity, promoting the metastasis of lung and colorectal cancers [63,64].

Based on our results, we suggest that downregulated levels of NF-κB significantly contribute to reduced migration capacity and decreased MMP-2/-9 expression [65,66]. Although ROS may serve as a strong activator of NF-κB expression and some studies identified the ROS–NF-κB axis as a crucial signaling pathway in MMP-9 expression and activation [67,68], these effects were not recorded in the treatment with FUAgNP and SVAgNP. The results of our study indicate that a prooxidative environment did not provoke the activation of NF-κB, but conversely—the tested nanoparticles induced the drop in NF-κB levels. We propose that alternative signaling pathways in HCT-116 cells are activated in response to FUAgNP and SVAgNP exposure, primarily due to the strong nitric oxide production and the activation of Nrf-2, with the rise of both parameters recorded in the study.

Nrf-2 and NF-κB exhibit complex interplay, crucial for modulating the inflammatory response and protecting cells from oxidative stress. Nrf-2 inhibits NF-κB activation by increasing the level of antioxidants and cytoprotective enzymes, which decreases the level of NF-κB, simultaneously preventing IκB-α degradation, a strong inhibitor of NF-κB, hindering NFκB-dependent transcription. The activation of Nrf-2 provoked by prooxidative effects of the tested nanoparticles could be the considerable underlying mechanisms of the inhibition of the NF-κB-MMP-9 axis detected in our study. Furthermore, NF-κB is an upstream regulator of inducible COX-2 expression, which activates the production of various inflammation mediators.

COX-2 levels were significantly reduced following the treatment with both FUAgNP and SVAgNP, confirming the suppression of inflammatory signaling recorded in the levels of NF-κB. COX-2 overexpression is frequently linked with increased metastatic potential in colorectal tumors [14,69]. Similar suppression of the NF-κB/COX-2 axis has been reported for other plant-derived AgNPs, supporting the concept that redox-mediated modulation of inflammatory signaling represents an important mechanism underlying the antimetastatic activity of silver biogenic nanoparticles [70,71].Various studies indicate that NO anti-inflammatory effects may be exerted via its ability to inhibit NF-κB since nitric oxide and NF-κB exhibit negative feedback crosstalk due to the regulative balance loop of inflammation [72]. Since the concentrations of NO in our study were significantly elevated compared to non-treated cells, we propose that this effect could be an additional/synergistic mechanism, next to Nrf-2-induced downregulation of NF-κB, and consequently the expression of COX-2-dependent inflammatory mediators.

The variability in biological activity of the green-synthesized AgNPs has been widely attributed to differences in the phytochemical composition of the plant extracts used during nanoparticle synthesis [73,74,75,76,77]. Although several studies have investigated AgNPs synthesized using *Salvia* species, data regarding *Filipendula ulmaria*-derived AgNPs remain limited. The present findings, together with previous studies in breast cancer models, support the conclusion that FUAgNP and SVAgNP exert antitumor and antimetastatic effects through the coordinated modulation of oxidative stress, inflammatory signaling, transcriptional regulation, and inhibition in proteolytic activities [23].

Overall, the obtained results demonstrate that FUAgNPs and SVAgNPs exert anti-inflammatory and anti-invasive effects in HCT-116 colorectal cancer cells through the coordinated modulation of nitric oxide production, Nrf-2-mediated redox responses, and downregulation of the NF-κB/COX-2 inflammatory pathway. These findings suggest that disruption of redox homeostasis represents an important mechanism underlying the ability of green-synthesized AgNPs to attenuate migration-associated and inflammatory responses in colorectal cancer cells. Therefore, FUAgNPs and SVAgNPs may represent promising candidates for further investigation as complementary nanotherapeutic approaches targeting colorectal cancer progression and metastasis.

## 5. Conclusions

In this study, we elucidated the potential underlying mechanisms of the antimetastatic and anti-inflammatory activity of two green-synthesized silver nanoparticles, FUAgNP and SVAgNP, providing a strong rationale for their further investigation in nano-based anticancer therapies, particularly in antimetastatic strategies. Both nanoparticles exhibited a significant inhibition of the migratory and invasion capacity of HCT-116 colorectal cancer cells via nitric oxide signaling modulation, as well as considerable anti-inflammatory effects based on the NF-κB-COX-2 axis. The proposed mechanism involves ROS/NO-mediated disruption of redox homeostasis, activation of Nrf-2-dependent antioxidant responses, and the suppression of NF-κB/COX-2 signaling, collectively streaming to the downregulation of MMP-2 and MMP-9 levels and activity. The ability of these plant-derived nanoparticles to disrupt key regulatory pathways involved in tumor invasion and dissemination supports their potential as effective and potentially safer anticancer nanomaterials. Furthermore, plant extract-mediated synthesis provides an eco-friendly production method, offering additional translational benefit. These findings underscore the therapeutic potential of FUAgNP and SVAgNP in colorectal cancer and their relevance as multifunctional nanotherapeutics capable of targeting multiple cancer hallmarks. Future investigations should focus on in vivo validation, including pharmacokinetics, biodistribution, long-term safety, and potential synergistic effects with standard chemotherapeutic regimens, to fully define their clinical applicability and translational value.

## Data Availability

The data supporting the findings of this study are available from the corresponding authors upon reasonable request.

## Abbreviations

The following abbreviations are used in this manuscript:

AgNPs	Silver Nanoparticles
FUAgNP	Aqueous Extract of *Filipendula ulmaria*
SVAgNP	Aqueous Extract of *Salvia verticillata*
ROS	Reactive Oxygen Species
RNS	Reactive Nitrogen Species
MMP-2	Matrix Metalloproteinases-2
MMP-9	Matrix Metalloproteinases-9
Nrf-2	Nuclear Factor Erythroid 2-Related Factor 2
NF-κB	Nuclear Factor Kappa-Light-Chain-Enhancer of Activated B Cells
DMEM	Dulbecco’s Modified Eagle Medium
FBS	Fetal Bovine Serum
NBT	Nitro Blue Tetrazolium
DMSO	Dimethyl Sulfoxide
NO_2_^−^	Nitrite
NO	Nitric Oxide
TME	Tumor Microenvironment
ONOO^−^	Peroxynitrite
